# FARO server: Meta-analysis of gene expression by matching gene expression signatures to a compendium of public gene expression data

**DOI:** 10.1186/1756-0500-4-181

**Published:** 2011-06-11

**Authors:** Mieszko P Manijak, Henrik B Nielsen

**Affiliations:** 1 Center for Biological Sequence Analysis, Department of Systems Biology, Technical University of Denmark, Kemitorvet, Building 208, DK-2800 Lyngby, Denmark

## Abstract

**Background:**

Although, systematic analysis of gene annotation is a powerful tool for interpreting gene expression data, it sometimes is blurred by incomplete gene annotation, missing expression response of key genes and secondary gene expression responses. These shortcomings may be partially circumvented by instead matching gene expression signatures to signatures of other experiments.

**Findings:**

To facilitate this we present the Functional Association Response by Overlap (FARO) server, that match input signatures to a compendium of 242 gene expression signatures, extracted from more than 1700 *Arabidopsis *microarray experiments.

**Conclusions:**

Hereby we present a publicly available tool for robust characterization of *Arabidopsis *gene expression experiments which can point to similar experimental factors in other experiments. The server is available at http://www.cbs.dtu.dk/services/faro/.

## Findings

Often gene expression studies identify more differentially expressed genes than can readily be functionally analyzed in follow up experiments. Fortunately, some of these genes typically are annotated either directly or through sequence similarity to other annotated genes, helping the scientist to interpret the observed transcriptional response. In many cases the transcripts can even be annotated with controlled vocabularies like the Gene Ontology [1] or Kyoto Encyclopedia of Genes and Genomes [2], facilitating systematic annotation analysis. Numerous successful examples of this type of analysis are found in the literature [3]. However, this type of analysis depends on a high coverage of annotated genes that respond transcriptionally to stimulus. Alternatively, meta-analysis of gene expression data can identify experimental conditions that result in similar transcriptional responses. This type of analysis has been done in a series of organisms, for example in yeast [4], in Human cell lines [5] and in Arabidopsis thaliana [6]. A key utilization of this type of analysis is in mutant, disease and drug characterization and matching.

### Algorithm

Here we present a web-based implementation of the Functional Association by Response Overlap (FARO) [6] approach allowing comparison of a user provided gene expression signature, against a pre-compiled compendium. The approach matches the transcriptional response based on the identity and the response direction (over or under expressed) of the differentially expressed genes, ignoring the magnitude of the response. Previously, we demonstrated that this simplistic approach largely overcomes experimental biases and allows reliable comparison between experiments conducted under varying conditions in different laboratories and at the same time is simple enough to allow human interpretations of the results [6]. The approach gains most of its robustness from avoiding direct comparison between the measurements in different experiments and instead comparing outcomes of comparisons between contrasts contained within a experimental design. Hence, the between experiment similarity measure is the number of intersecting genes between lists of differentially expressed genes from two experiments. In addition, congruence of the gene expression response direction adds important insight into the nature of the signature comparison.

### Testing

Since the web server implementation of the FARO approach based on the script prepared and tested in the original experiment [6] testing was restricted to processing data submitted via the web site by a user. The server was tested with data files containing mixed Affymetrix ATH1 probe identifiers and AGI locus identifiers as well as unknown identifiers and empty lines. Tests proved that the implementation handles all mentioned cases correctly.

### Implementation

The FARO server allows the user to compare an expression signature against a compendium of signatures. The latter consists of 242 experimental signatures defined by the top 1209 differentially expressed genes. 1209 is the median number genes being significant across the compendium at significance level of 0.05. The experimental factors were extracted from more than 1700 public microarray experiments. The experimental factors represent various conditions and perturbations that are described in details on the server webpage. The server accepts a table containing at least 50 identifiers of either Affymetrix ATH1 probe set or AGI locus identifiers and compares the query gene list to the FARO compendium at the probe set level. For full functionality the input table must contain two columns containing identifiers and response direction as indicated by a signed number (possibly the log fold change), respectively. Optionally, the response direction may be indicated by "+" or -", or alternatively left out entirely. In the latter case the congruence analysis is omitted.

The comparison returns a list of associated experimental factors that are filtered according to user specified thresholds. Two options are available for setting the threshold:

1. The Overlap percentage threshold returns associated factors that have overlap with the query list that equals or exceeds the indicated percentage. Here the percentage means the percentage of the query length.

2. The Rank threshold returns the r factors with the strongest overlap to the query list. Where the user specifies the rank (r) threshold.

**Figure 1:**
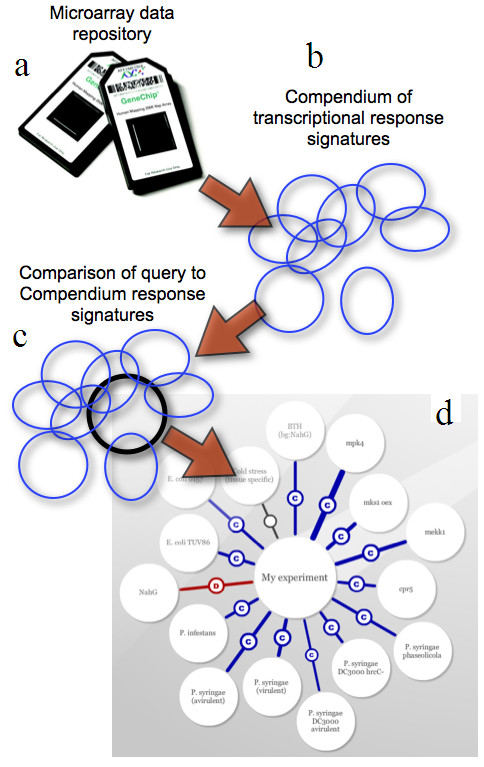
**Workflow of the FARO server**. (A) Microarray expression data was extracted and processed in order to acquire (B) response signature compendium (a collection of top ranking differentially expressed genes lists). (C) Query signature is compared to the compendium resulting in a set of possible functional associations. (D) A graph representation of the functional associated factors. Edge thickness indicates the association strength and the coloration represents significant response direction congruence (blue) or dissimilarity (red). Gray edges indicate insignificant directionality.

The server output is given as a table of functional associated compendium factors. For each factor a list of overlapping TAIR annotated gene identifiers is given. The table of associated factors furthermore contains statistics on the overlap including the number of overlapping genes, the p-value (Fisher's exact test), the response direction similarity (congruence) and the significance of the congruence estimated by binomial statistics. Furthermore, the FARO server visualizes the query experiment in the context of a dynamic graph displaying the association network of all compendium factors (as seen in Figure 1D). The dynamic graph enables the user to navigate through the association space to deepen the understanding of the individual factors meaning.

### Availability and Requirements

Project name: FARO server

Project home page: http://www.cbs.dtu.dk/services/faro/

Operating system(s): Platform independent

Programming language: Perl

Other requirements: None

License: GNU GPL.

Any restrictions to use by non-academics: none

## Competing interests

The authors declare that they have no competing interests.

## Authors' contributions

HBN conceived and implemented the original algorithm, MPM adapted the original implementation for web server purposes and implemented the web server and performed tests. Both authors wrote and approved the final manuscript.
